# Supplementary material for: Bayesian refinement of protein structures and ensembles against SAXS data using molecular dynamics
Source: PLoS Comput Biol. 2017 Oct 18;13(10):e1005800. doi: 10.1371/journal.pcbi.1005800 (PMC5662244; doi:10.1371/journal.pcbi.1005800)
Supplement: S1 Archive — (BZ2) [file pcbi.1005800.s011.tar.bz2 › gromacs_4.62_bayesian_saxs_refi/share/html/online/g_nmeig.html]

g\_nmeig


| g\_nmeig Main Table of Contents |

**VERSION 4.6  
Sat 19 Jan 2013**

---

### Description

g\_nmeig calculates the eigenvectors/values of a (Hessian) matrix,
which can be calculated with mdrun.
The eigenvectors are written to a trajectory file (-v).
The structure is written first with t=0. The eigenvectors
are written as frames with the eigenvector number as timestamp.
The eigenvectors can be analyzed with g\_anaeig.
An ensemble of structures can be generated from the eigenvectors with
g\_nmens. When mass weighting is used, the generated eigenvectors
will be scaled back to plain Cartesian coordinates before generating the
output. In this case, they will no longer be exactly orthogonal in the
standard Cartesian norm, but in the mass-weighted norm they would be.

This program can be optionally used to compute quantum corrections to heat capacity
and enthalpy by providing an extra file argument -qcorr. See the GROMACS
manual, Chapter 1, for details. The result includes subtracting a harmonic
degree of freedom at the given temperature.
The total correction is printed on the terminal screen.
The recommended way of getting the corrections out is:

g\_nmeig -s topol.tpr -f nm.mtx -first 7 -last 10000 -T 300 -qc [-constr]

The -constr option should be used when bond constraints were used during the
simulation **for all the covalent bonds**. If this is not the case,
you need to analyze the quant\_corr.xvg file yourself.

To make things more flexible, the program can also take virtual sites into account
when computing quantum corrections. When selecting -constr and
-qc, the -begin and -end options will be set automatically as well.
Again, if you think you know it better, please check the eigenfreq.xvg
output.

### Files

| option | filename | type | description |
| --- | --- | --- | --- |
| **-f** | hessian.mtx | Input | Hessian matrix |
| **-s** | topol.tpr | Input | Run input file: tpr tpb tpa |
| **-of** | eigenfreq.xvg | Output | xvgr/xmgr file |
| **-ol** | eigenval.xvg | Output | xvgr/xmgr file |
| **-os** | spectrum.xvg | Output, Opt. | xvgr/xmgr file |
| **-qc** | quant\_corr.xvg | Output, Opt. | xvgr/xmgr file |
| **-v** | eigenvec.trr | Output | Full precision trajectory: trr trj cpt |

### Other options

| option | type | default | description |
| --- | --- | --- | --- |
| **-[no]h** | bool | no | Print help info and quit || **-[no]version** | bool | no | Print version info and quit || **-nice** | int | 19 | Set the nicelevel || **-xvg** | enum | xmgrace | xvg plot formatting: xmgrace, xmgr or none || **-[no]m** | bool | yes | Divide elements of Hessian by product of sqrt(mass) of involved atoms prior to diagonalization. This should be used for 'Normal Modes' analysis || **-first** | int | 1 | First eigenvector to write away || **-last** | int | 50 | Last eigenvector to write away || **-maxspec** | int | 4000 | Highest frequency (1/cm) to consider in the spectrum || **-T** | real | 298.15 | Temperature for computing quantum heat capacity and enthalpy when using normal mode calculations to correct classical simulations || **-[no]constr** | bool | no | If constraints were used in the simulation but not in the normal mode analysis (this is the recommended way of doing it) you will need to set this for computing the quantum corrections. || **-width** | real | 1 | Width (sigma) of the gaussian peaks (1/cm) when generating a spectrum |

---

http://www.gromacs.org  
gromacs@gromacs.org
